# Strategies and delivery systems for cell-based therapy in autoimmunity

**DOI:** 10.3389/fddev.2024.1436842

**Published:** 2024-08-08

**Authors:** Matteo Puccetti, Claudio Costantini, Aurélie Schoubben, Stefano Giovagnoli, Maurizio Ricci

**Affiliations:** ^1^ Department of Pharmaceutical Sciences, University of Perugia, Perugia, Italy; ^2^ Department of Medicine and Surgery, University of Perugia, Perugia, Italy

**Keywords:** immunotherapy, antigen-presenting cells, autoimmunity, genetic engineering, nanoparticles

## Abstract

This review article explores the potential of engineering antigen-presenting cells (APCs) for the immunotherapy of autoimmune diseases. It discusses various strategies for modifying APCs to induce antigen-specific tolerance, thereby mitigating autoimmune responses. The review covers recent advancements in APC engineering techniques, including genetic modification and nanoparticle-based approaches, and evaluates their efficacy in preclinical models and clinical trials. Additionally, challenges and future directions for the development of APC-based immunotherapies for autoimmunity – and other forms of cell-based immunotherapy – are discussed. Along this direction, this review (*i*) describes various strategies for engineering APCs, including genetic modification, nanoparticle delivery systems, and *ex vivo* manipulation techniques; (*ii*) discusses the selection of target antigens and the design of APC-based immunotherapies, and (*iii*) reviews preclinical models used to evaluate the efficacy and safety of engineered APCs in inducing antigen-specific tolerance.

## 1 Introduction

In the realm of immunotherapy, the pursuit of precision and efficacy stands as a beacon of hope for combating pathogenic immunity, especially in the context of autoimmune diseases and chronic inflammatory conditions. Traditional approaches often entail systemic administration of immunomodulatory agents, risking off-target effects and diminishing therapeutic outcomes. However, the advent of engineering antigen-presenting cells (APCs) presents a paradigm shift towards a more targeted and personalized therapeutic strategy (Strzelec et al., 2023; Chasov et al., 2024; Hassan et al., 2024).

Central to this endeavor is the concept of leveraging APCs as vehicles for the optimal delivery of immunomodulatory biologicals. By harnessing the innate antigen-presenting capabilities of dendritic cells, macrophages, and B cells, researchers aim to finely tune the immune response, mitigating aberrant activation while preserving protective immunity. Through precise manipulation of APCs, either *ex vivo* or *in situ*, it becomes possible to tailor the delivery of therapeutic payloads to specific disease targets, thereby minimizing systemic toxicity and maximizing therapeutic efficacy (Fucikova et al., 2019).

This review aims to focus on the multifaceted landscape of engineering APCs for the optimal delivery of immunomodulatory biologicals in pathogenic immunity. We will explore the diverse array of engineering strategies, ranging from genetic modification and nanoparticle-based approaches to exosome-mediated delivery systems. Furthermore, we will examine the potential applications of engineered APCs across a spectrum of autoimmune diseases, chronic inflammatory conditions, and transplant rejection scenarios.

As we embark on this journey, we recognize the inherent challenges and complexities that lie ahead. From fine-tuning the immunomodulatory payload to navigating the intricate interplay of immune cell subsets within the diseased microenvironment, each step presents both opportunities and obstacles. Nevertheless, by synergizing cutting-edge technologies with deepening insights into immune regulation, we aspire to chart a course towards precision immunotherapy, where the delivery of therapeutic biologicals by engineered APCs stands poised to redefine the treatment landscape of pathogenic immunity. Our major goals are: (*i*) to provide an overview of autoimmune diseases and their underlying mechanisms are: (*ii*) to highlight the limitations of current treatments, such as non-specific immunosuppression and potential side effects; and (*iii*) to introduce the concept of antigen-specific tolerance induction using engineered APCs as a promising therapeutic approach for autoimmunity.

## 2 A step beyond conventional treatments

Antigen-specific immunotherapies hold significant promise for enhancing the precision of autoimmune disease treatments, which currently rely solely on broad, nonspecific immunosuppression. A critical aspect of antigen-specific immunotherapy involves delivering autoantigens, akin to the methods used in allergy desensitization. Although clinical success in allergen-specific tolerance has been achieved for over a century, no FDA-approved antigen-specific immunotherapy exists yet. The substantial differences in the physicochemical properties of allergens and autoantigens affect their interactions with the immune system. Approved allergen-specific therapies are generally water-soluble, neutrally charged protein fractions ranging from 10 to 70 kDa. In contrast, autoantigens are native proteins with diverse sizes, solubilities, and charges, making them prone to immunogenicity. To adapt the successful strategies of allergen desensitization to antigen-specific immunotherapy, innovative delivery methods are needed to properly format autoantigens, direct their biodistribution, and activate the appropriate immune responses (Griffin et al., 2020; Song et al., 2020; Benne et al., 2022; Li et al., 2024).

Systemic autoimmune diseases, including systemic lupus erythematosus, systemic sclerosis, and rheumatoid arthritis, are characterized by dysregulation in both the innate and adaptive immune systems. These conditions share common pathogenic features, such as the interferon signature, loss of self-tolerance to nuclear antigens, and increased tissue damage like necrosis and fibrosis. First-line treatments typically involve glucocorticoids and immunosuppressants, which have limited specificity and can lead to tolerance issues.

A range of new immunotherapies has been developed, targeting cellular and soluble factors involved in disease pathogenesis. These include monoclonal and bispecific antibodies, as well as other biological agents aimed at B cells, co-stimulatory molecules, cytokines or their receptors, and signaling molecules. Many of these new treatments have shown promising results in clinical trials. Chimeric antigen receptor (CAR)-T cell therapy is emerging as a highly promising approach for treating autoimmune diseases, with recent successes noted in systemic lupus erythematosus and systemic sclerosis. While CAR-T cell therapy and other cellular immunotherapies are currently more established in oncology, significant strides have also been made in treating autoimmune conditions. Advances in the implementation of cell-based immunotherapies have made these treatments more affordable for both cancer and autoimmune diseases (Ganeeva et al., 2022). The latest developments in cell-based immunotherapies are expected to revolutionize the treatment of autoimmune diseases (Chasov et al., 2024).

## 3 The nature of dendritic cells as antigen-presenting cells

**FIGURE 1:**
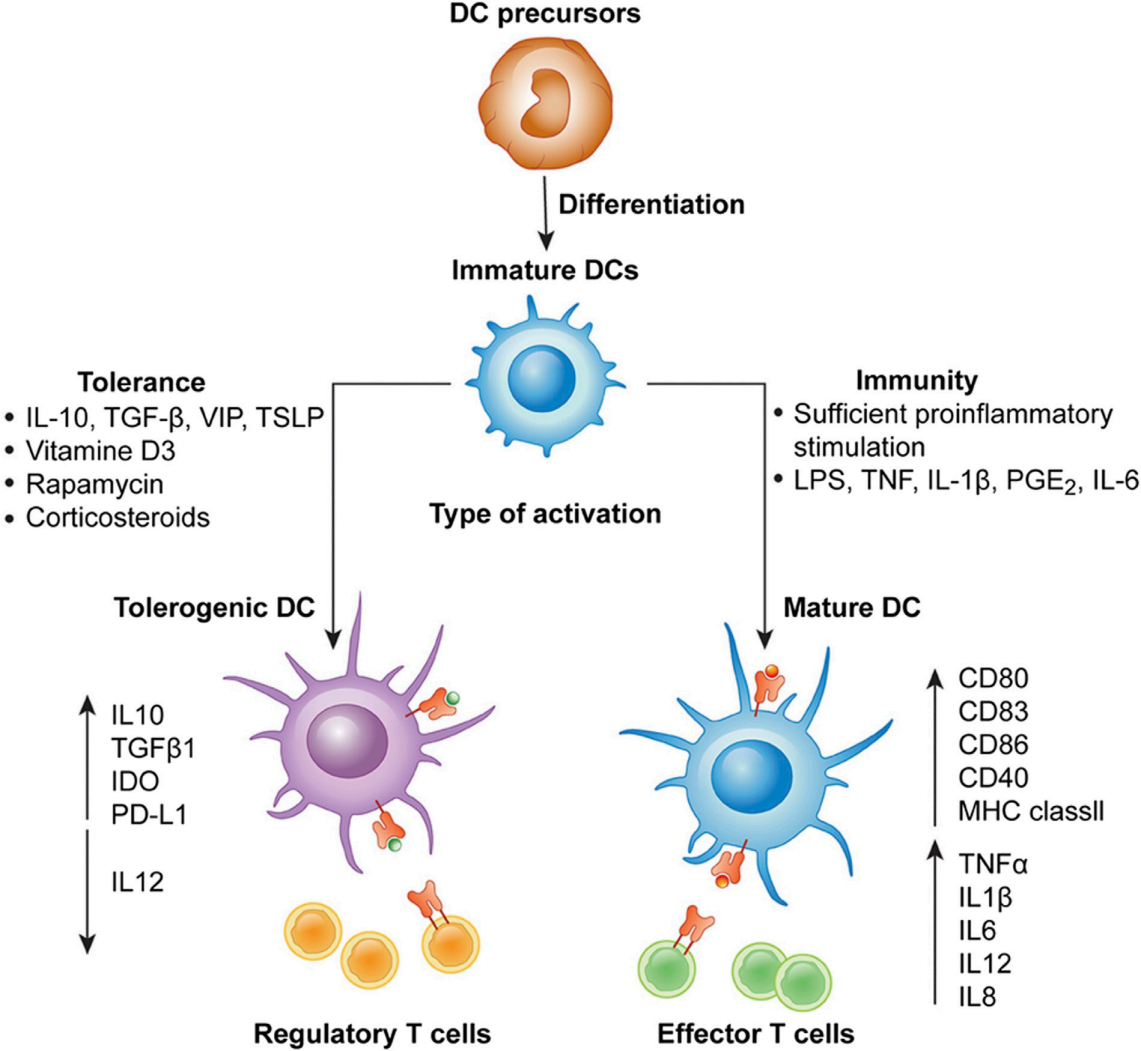
Differentiation of monocyte-derived activated vs. tolerogenic dendritic cells. Dendritic cells (DCs) differentiate from DC precursors into immature DCs (iDCs) in the presence of IL-4 and GM-CSF. In the presence of a maturation signal (proinflammatory cytokines and Toll-like receptor ligands), DCs become activated and transition to a stimulatory phenotype, which subsequently leads to the induction of effector/cytotoxic T cell responses. In contrast, incubation of iDCs with different mediators or genetic modification of DCs in the absence of maturation factors can lead to the generation of tolerogenic DCs, which induce anergy, apoptosis or activation of Tregs. Taken in whole from Ref (Fucikova et al., 2019). (Open Access under CC BY 4.0 DEED License.)

In 1868, Paul Langerhans discovered cells in the skin that resembled nerves due to their shape and reaction to gold salt. These cells puzzled scientists until 1973 when Ralph Steinman identified them as dendritic cells (DCs), a subset of which are Langerhans cells (LCs). DCs, important for activating CD4^+^ T-cell responses, travel from tissues to lymphoid organs when exposed to pathogens. As they migrate, they mature, increasing expression of molecules like MHC and CD80/CD86. DCs vary in origin, appearance, and function, initially classified as conventional (cDCs) and plasmacytoid (pDCs) dendritic cells. Later research showed both types can arise from myeloid or lymphoid precursors, though they mostly originate from myeloid precursors.Immunogenic DCs stimulate effector T cells, while tolerogenic DCs promote regulatory T cells. DC-T cell communication relies on three signals, disruption of which can lead to tolerogenic DC generation. Tolerogenic DCs have reduced costimulatory molecules and increased inhibitory receptors, along with altered cytokine production. DCs have been explored for treating diseases like HIV-1, cancers, graft versus host disease (GVHD), and autoimmune diseases (Iberg et al., 2017). Clinical trials show promise, but standardizing therapeutic DC production and delivery remains a challenge (Worbs et al., 2017; Collin and Bigley, 2018; Balan et al., 2019; Yin et al., 2021; Heras-Murillo et al., 2024) DCs play a critical role in regulating immune responses by either promoting immunity (immunogenic) or inducing tolerance (tolerogenic). Several factors influence the immunogenic vs. tolerogenic function of DCs (Figure 1).

Thus, in general, the major factors influencing the functional phenotype of DCs are:

I. **Maturation Status**: Immature DCs are more likely to induce tolerance, while mature DCs are more immunogenic. Maturation can be influenced by various factors such as microbial products, inflammatory cytokines, and tissue microenvironment (Dudek et al., 2013; Nam et al., 2021).
II. **Microbial Products**: Pathogen-associated molecular patterns (PAMPs) and danger-associated molecular patterns (DAMPs) can activate DCs and promote immunogenic responses. In contrast, certain microbial products or their derivatives can induce tolerogenic responses, leading to immune suppression or tolerance (Eppensteiner et al., 2019; Wilson et al., 2022).
III. **Cytokine Milieu**: The cytokine environment in which DCs mature or function greatly influences their phenotype and function. For example, interleukin-10 (IL-10) promotes DC tolerogenicity, while interleukin-12 (IL-12) promotes DC immunogenicity (Schülke, 2018).
IV. **Tissue Microenvironment**: The tissue microenvironment provides signals that instruct DCs to adopt specific phenotypes. For instance, the presence of regulatory T cells (Tregs) or anti-inflammatory cytokines can skew DCs towards a tolerogenic phenotype (Plesca et al., 2022).
V. **Toll-like Receptor (TLR) Signaling**: Engagement of TLRs on DCs by microbial ligands can lead to either immunogenic or tolerogenic responses, depending on the specific TLR and context (Iberg and Hawiger, 2020).
VI. **Metabolic State**: Metabolic cues can influence DC function. For example, a shift towards glycolysis is associated with immunogenic DCs, whereas oxidative phosphorylation is associated with tolerogenic DCs (Wculek et al., 2019).
VII. **Costimulatory Molecules**: Expression of costimulat ory molecules such as CD80, CD86, and CD40 on DCs can influence their ability to induce T cell activation or tolerance (Grohmann et al., 2002; Orabona et al., 2004). As one example, CTLA-4-immunoglobulin (CTLA-4–Ig) can alter the functional phenotype of immunogenic dendritic cells in experimental type-1 diabetes by modulating tryptophan catabolism. This study demonstrates that long-term survival of pancreatic islet allografts, facilitated by CTLA-4–Ig, relies on effective tryptophan catabolism in the host. *In vitro*, CTLA-4–Ig influences cytokine-dependent tryptophan catabolism in B7-expressing dendritic cells. This indicates that CTLA-4’s role *in vivo* includes regulating tryptophan catabolism and acting as a ligand for B7 receptor molecules, which transduce intracellular signals, thus contributing to peripheral tolerance (Grohmann et al., 2002).
VIII. **Induced Inversion of Functional Phenotype**: Inducing the expression of tolerogenic enzymes like IDO1 (indoleamine 2,3-dioxygenase 1) in dendritic cells is a strategy employed in immunology to promote immune tolerance (Grohmann et al., 2003). Dendritic cells are potent antigen-presenting cells that play a crucial role in initiating and regulating immune responses. IDO1 is involved in the degradation of the amino acid tryptophan, leading to the production of kynurenine. This metabolic pathway has immunomodulatory effects, including the suppression of T cell responses and the promotion of regulatory T cell (Treg) differentiation. By upregulating IDO1 expression in dendritic cells, researchers aim to create an immunosuppressive microenvironment that helps to dampen immune responses and induce tolerance, particularly in settings of autoimmunity, transplantation, or allergy. Several approaches can be used to induce IDO1 expression in dendritic cells, including the use of pharmacological agents, genetic manipulation, or stimulation with specific cytokines or signaling pathways known to regulate IDO1 expression (Bessede et al., 2014). This strategy holds promise for the development of novel therapies aimed at modulating immune responses and promoting tolerance in various immune-mediated diseases (Gargaro et al., 2022).

Therapeutically, tipping the balance of DC function towards either immunogenicity or tolerogenicity holds promise for treating various human diseases. Immunogenic DCs can be generated *ex vivo* and used as cancer vaccines to stimulate anti-tumor immune responses (Nava et al., 2021). Tolerogenic DCs can be exploited to induce antigen-specific tolerance in autoimmune diseases like rheumatoid arthritis, multiple sclerosis, and type 1 diabetes (Nava et al., 2021). Tolerogenic DCs can be used to promote immune tolerance in organ transplantation, reducing the need for immunosuppressive drugs and the risk of rejection. Tolerogenic DCs can be harnessed to induce immune tolerance in allergic diseases and asthma (Morianos and Semitekolou, 2020). Finally, modulating DC function towards immunogenicity can enhance host immune responses against pathogens, while inducing tolerogenic DCs may help in controlling excessive inflammation in chronic infections (Manicassamy and Pulendran, 2011).

Recent literature has expanded our understanding of strategies for manipulating DC phenotype revealing a metabolic communication pathway involving IDO1-expressing dendritic cells (cDC1), which extends their immunoregulatory capacity to cDC2 subset through the production of the tryptophan metabolite l-kynurenine, an activating ligand for Aryl hydrocarbon Receptor (AhR) (Fallarino et al., 2014; Grohmann and Puccetti, 2015). This pathway plays a crucial role in maintaining tolerance and preventing autoimmune diseases (Gargaro et al., 2022; Guo et al., 2023). These studies further suggest that targeting this metabolic axis could be a potential therapeutic strategy for treating autoimmune demyelinating diseases. Another significant avenue of exploration involves the refinement of gene editing techniques to enhance the functionality and specificity of APCs, particularly conventional dendritic cells (cDCs) and tolerogenic dendritic cells. By understanding the factors that influence the function of DCs and manipulating them accordingly, it is possible to develop novel therapeutic strategies for a wide range of human diseases (Hilkens and Isaacs, 2013; Passeri et al., 2021).

## 4 Strategies for engineering antigen-presenting cells

Advancements in gene editing tools, such as clustered regularly interspaced short palindromic repeats/associated protein 9 (CRISPR-Cas9) and base editing systems, offer unprecedented precision in modifying the genome of APCs. These techniques enable researchers to precisely manipulate key molecular pathways and signaling cascades within APCs, thereby enhancing their antigen presentation capabilities and modulating their immune-regulatory functions. By engineering APCs to overexpress or downregulate specific genes involved in antigen processing, presentation, and immune regulation, researchers aim to tailor the immunogenic or tolerogenic properties of APCs according to the desired therapeutic outcome (Elsayed et al., 2023). Furthermore, recent studies have focused on optimizing the delivery and expression of immunomodulatory factors within APCs to promote immune tolerance and suppress unwanted immune responses. This includes the use of viral vectors, nanoparticles (NPs) (Cifuentes-Rius et al., 2021), or exosome-based delivery systems to efficiently deliver therapeutic genes or molecules into APCs *in vivo*. By precisely controlling the expression levels and kinetics of immunomodulatory factors within APCs, researchers aim to fine-tune the induction of antigen-specific immune tolerance while minimizing off-target effects (Zulfiqar et al., 2017; Allemailem et al., 2022; Li et al., 2023; Vashist et al., 2023; Adhikary et al., 2024).

CRISPR/Cas9 technology allows for precise editing of the genome to enhance the functionality of APCs. This can involve knocking out inhibitory molecules or inserting genes that enhance antigen presentation. Mechanistic Insight—CRISPR/Cas9 introduces double-strand breaks at specific genomic locations. The cell’s repair machinery, typically through non-homologous end joining (NHEJ) or homology-directed repair (HDR), repairs these breaks. This mechanism can be harnessed to knock out genes (by NHEJ) or introduce new genes (by HDR). Key Study—A pivotal study by Hsu et al. (Hsu et al., 2014) demonstrated the efficient editing of the PD-L1 gene in dendritic cells using CRISPR/Cas9, enhancing their ability to stimulate T-cells. Knockout of PD-L1 reduced the immunosuppressive signals and improved T-cell activation *in vitro* and *in vivo*, showing potential for enhancing cancer immunotherapy.

In addition to genetic engineering approaches, recent literature has also explored the use of biomaterial-based strategies for modulating APC function and behavior. Biomaterials, such as synthetic polymers, hydrogels, and scaffolds, can be engineered to mimic the native extracellular matrix and provide a microenvironment conducive to APC survival, migration, and interaction with immune cells (Yang et al., 2020; Jacob et al., 2021; Joyce et al., 2021; Fisher et al., 2022; Ren et al., 2023). By incorporating bioactive molecules, such as cytokines, growth factors, or immunomodulatory drugs, into these biomaterial platforms, researchers can spatially and temporally control the presentation of immunomodulatory signals to APCs, thereby directing immune responses towards tolerance induction or immune suppression.

Moreover, recent studies have highlighted the importance of considering the heterogeneity and plasticity of APC populations in the design of engineered APC-based therapies. By characterizing the phenotypic and functional diversity of APC subsets within different tissues and disease contexts, researchers can tailor their engineering strategies to target specific APC populations involved in the initiation or perpetuation of immune-mediated pathologies. This personalized approach holds promise for maximizing the therapeutic efficacy of engineered APC-based immunotherapies while minimizing off-target effects and adverse reactions (McCoach and Bivona, 2019; Brown et al., 2023; Lee-Chang and Lesniak, 2023).

Overall, the above literature data underscore the potential of engineering APCs as a versatile platform for modulating immune responses and treating a wide range of immune-mediated diseases, including autoimmunity, allergy, and transplant rejection. By leveraging advances in gene editing technologies, biomaterial science, and our understanding of APC biology, researchers are poised to develop next-generation APC-based immunotherapies with improved safety, efficacy, and specificity (Mashayekhi et al., 2024; Slezak et al., 2024).

### 4.1 Genetic modification

In recent times, tolerogenic DC treatment in clinical trials has demonstrated its safety, signaling a new phase in cell-based immunotherapy for conditions like autoimmunity and transplant rejection. Nonetheless, for tolerogenic DC therapy to become the preferred option, methods to boost their effectiveness must be explored (Streeter and Wraith, 2021). Whether tolerogenic DCs constitute a separate lineage or simply an activation state of conventional DCs remains uncertain. Specific signaling pathways and transcriptional programs, like those governed by Stat3, AhR, Socs2, and other signaling pathways may dictate the tolerogenic phenotype (Cheng et al., 2024). Hence, comprehending how tolerogenic DCs develop tolerance mechanisms at transcriptome, metabolome, and epigenome levels is vital (Lafita-Navarro et al., 2020; Barroso et al., 2021; Gargaro et al., 2021). Of particular interest, novel genetically glycoengineered human dendritic cell model reveals regulatory roles of α2,6-linked sialic acids in DC activation of CD4^+^ T cells and response to TNFα (Tian et al., 2024).

Studies in animals have shown that gene editing techniques can confer immune tolerance and antigen specificity to tolerogenic DCs, necessitating the development of precise editing methods. Consequently, various clinical trials are underway to assess the safety and efficacy of gene-modified tolerogenic DCs in suppressing autoimmune responses, aiming for a deeper understanding of their interactions with other inflammatory cells. However, the use of specific gene-modified tolerogenic DCs may not be universally suitable for treating autoimmune diseases, necessitating evaluation of optimal dosages, infusion schedules, and immunosuppressive regimens tailored to each condition (Khalaf et al., 2020; Mansilla et al., 2023).

Another crucial aspect of gene-modified tolerogenic DC therapy is the identification of effective assays for monitoring efficacy and detecting potential adverse immune responses or signs of undesired activation. Ongoing clinical trials focusing on precise immune monitoring are expected to unveil efficacy biomarkers, essential for refining regulatory cell therapy to prevent organ transplant rejection and promote long-term tolerance.

Incorporating gene-editing technology represents the logical progression in advancing tolerogenic DC therapy, holding significant promise for addressing autoimmunity and transplant tolerance (Ma et al., 2024). Such gene-editing techniques include:

#### 4.1.1 Introduction of target antigens

APCs can be genetically engineered to express specific target antigens associated with autoimmune diseases or other pathological conditions. This approach ensures efficient presentation of disease-specific antigens to immune cells, promoting antigen-specific tolerance. The primary objective of treatment strategies for autoimmune and allergic disorders is to restore immunological tolerance to self-antigens or harmless environmental allergens (Streeter and Wraith, 2021). Among interventions, antigen-specific immunotherapy stands out for its proven ability to modify the course of disease, often leading to long-lasting remission in various allergic conditions. Emerging evidence suggests that by specifically targeting pathogenic T cells in autoinflammatory and autoimmune contexts, it becomes possible to restore immune balance between effector and regulatory cells, thus influencing the progression of the disease (Ghobadinezhad et al., 2022). Recent literature explores the pivotal insights gained from the development of antigen-specific immunotherapies and their potential implications for future interventions (Richardson and Wraith, 2021). With our current understanding and the advanced technology available for monitoring immune cell characteristics and activities, the achievement of targeted tolerance restoration seems increasingly probable, shifting the question from “if,” “when,” and “how” (Bevington et al., 2020). Route of administration appears key to targeted restoration of immunological tolerance to self-antigens or innocuous environmental antigens (Richardson and Wraith, 2021); (Box 1).

BOX 1 **Route of administration for tolerance induction.**

Tolerance induction through mucosal surfaces (such as oral, nasal, and sublingual routes) has been historically favored. These sites are constantly exposed to environmental antigens yet, in healthy individuals, do not trigger immune responses to them (Weiner et al., 2011).

Pioneering experiments by Weiner and his team in various animal models of autoimmune diseases demonstrated the significant effectiveness of orally administered antigens in preventing diseases (Thaventhiran et al., 2012). However, oral tolerance was notably less effective in animals already sensitized (which better simulate ongoing human diseases) (Streeter et al., 2015). Clinical trials attempting oral tolerance induction in multiple sclerosis (MS) using myelin basic proteins were safe but ineffective (Conde et al., 1998). This ineffectiveness is likely due to the low antigen doses used in patients compared to animal studies, as well as the generally weak immune responses towards autoantigens (Benson et al., 1999).

Even in allergic diseases, where antigens typically provoke strong immune responses, oral delivery of antigens doesn’t consistently achieve tolerance. An exception is peanut allergy, where repeated doses of pure peanut protein up to 800 mg reduced sensitivity after 30 weeks of treatment. However, the long-term efficacy and need for ongoing therapy were not evaluated post-treatment (Anagnostou et al., 2014). Directly delivering the offending antigen to the hypersensitive site might tap into natural regulatory feedback loops for disease modification. However, achieving significant protein amounts, especially with recombinant allergens, is costly and inefficient due to degradation in the stomach before reaching the gut.

Mucosal delivery via sublingual immunotherapy (SLIT) and systemic delivery via subcutaneous immunotherapy (SCIT) have proven clinically effective using much lower antigen doses and are now standard in allergen immunotherapy (Jutel et al., 2015; Jutel et al., 2016). While few studies directly compare SCIT and SLIT efficacy, their mechanisms of action likely differ slightly (Lawrence et al., 2016; Schulten et al., 2016).

Intralymphatic antigen delivery, though still in early stages, has shown remarkable efficacy in murine models and allergy clinical trials (Senti et al., 2008; Martínez-Gómez et al., 2009; Senti et al., 2012). Direct delivery of grass pollen allergen intralymphatically has resulted in safe, pain-free, and effective allergen-specific tolerance much quicker than standard SCIT therapy. It's expected that this approach could be applied to other allergies, and upcoming trials will be closely monitored.

In the realm of autoimmune diseases, pre-clinical investigations in mouse models have shown varying efficacy among delivery routes, with subcutaneous > intranasal > oral delivery (Burton et al., 2014). Clinical trials in diseases like relapsing-remitting MS and Graves' disease have shown significant decreases in disease severity with subcutaneous or intradermal delivery of tolerogenic peptides (Chataway et al., 2018; Pearce et al., 2019). Importantly, studies have indicated that soluble peptides are detected on the surface of specific cells within minutes of subcutaneous injection (Oldfield et al., 2001). Repeated delivery of soluble peptide induces IL-10 expression in anergic T cells, contributing to tolerance (Burton et al., 2014; Bevington et al., 2020).

In the DIAGNODE trial for autoimmune disease (Puente-Marin et al., 2023), the intralymphatic route was employed, involving direct injection of glutamic acid decarboxylase antigen into lymph nodes of type 1 diabetes patients, resulting in a promising reduction in insulin requirement post-treatment. While this route may offer more potent immune tolerance induction, it's less practical for maintenance therapy (Ludvigsson et al., 2008; Ludvigsson et al., 2017).

#### 4.1.2 Expression of regulatory molecules

Genetic modification enables the expression of regulatory molecules, such as cytokines (e.g., IL-10, TGF-β) or co-stimulatory molecules (e.g., PD-L1), on APCs. These molecules modulate the immune response by promoting regulatory T cell differentiation or inhibiting effector T cell activation, thereby inducing immune tolerance. In an interesting series of observations (Matoba et al., 2019), the Authors initially reported that regulatory T (Treg) cells expressing CTLA‐4 on the cell surface are abundant in head and neck squamous cell carcinoma (HNSCC). However, the role of expanded Treg cells in the tumor microenvironment of HNSCC remained unclear. In a subsequent study, they revealed that the tumor microenvironment of HNSCC is characterized by the high expression of genes related to Treg cells, DCs, and interleukin (IL)‐17‐related molecules. Increased expression of *IL17A*, *IL17F*, or *IL23A* contributed to a favorable prognosis of HNSCC. In the tumor microenvironment of HNSCC, *IL23A* and *IL12B* were expressed in mature DCs enriched in regulatory molecules (mregDCs). These mregDCs in HNSCC displayed a migratory and mature phenotype, with their signature genes strongly correlating with Treg signature genes in HNSCC. Additionally, *IL17A* was found to be highly expressed in Th17 cells and exhausted CD8^+^ T cells in HNSCC. These findings suggest that mregDCs in HNSCC may contribute to the prognosis by balancing Treg cells and effector T cells that produce IL‐17. The Authors concluded that targeting mregDCs could be a novel strategy for developing new immune therapies against HNSCC (Minohara et al., 2023).

#### 4.1.3 Gene editing technologies

**FIGURE 2:**
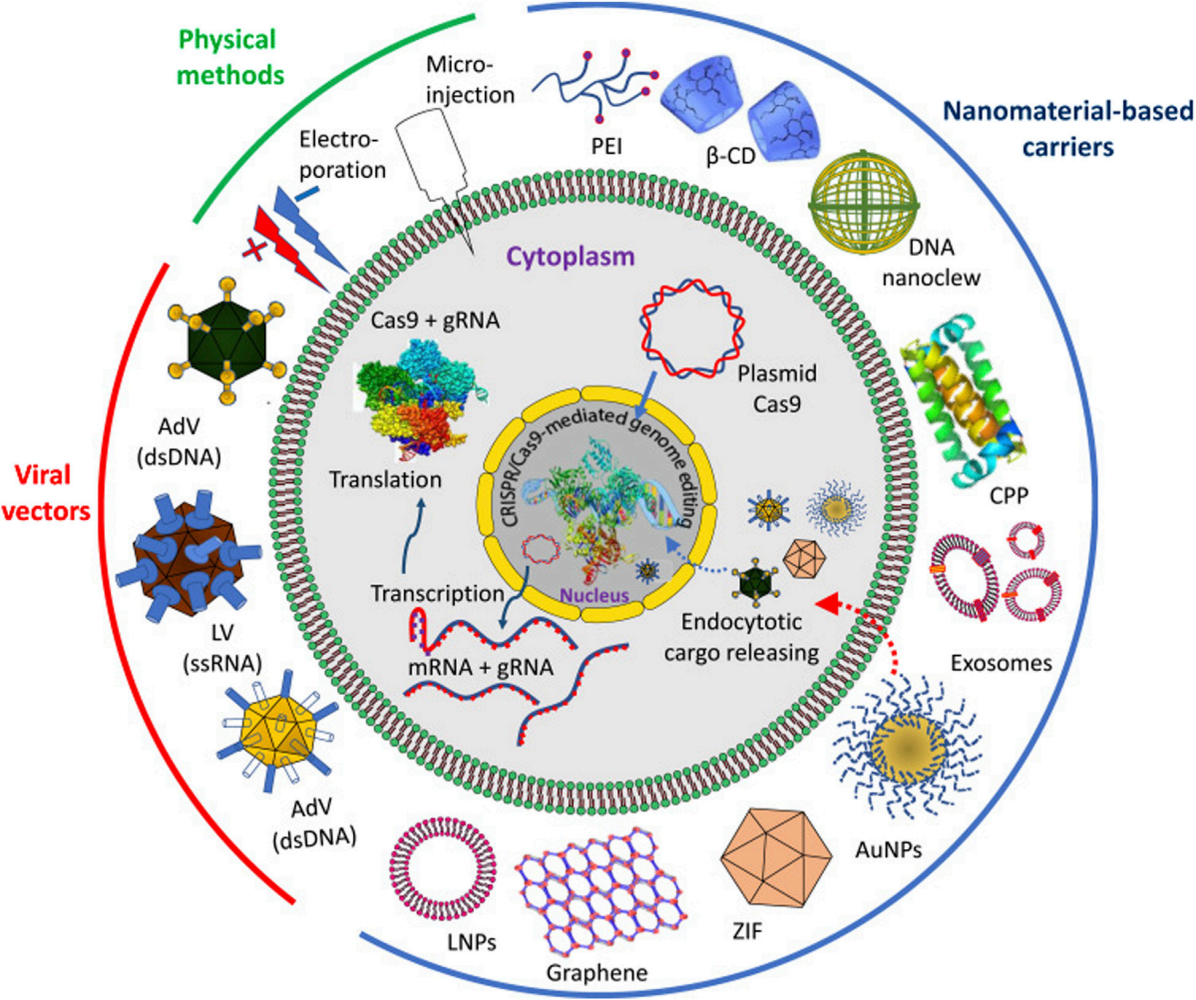
Delivery approaches and expression of CRISPR/Cas9 system *in vivo*. The delivery approaches can be through physical methods, viral vectors and by different nanomaterial-based carriers. Abbreviations: PEI, polyethyleneimine; β-CD, β-cyclodextrin; CPP, cell penetrating protein; AuNPs, gold nanoparticles; ZIF, zeolite imidazole; LNPs, lipid nanoparticles; AdV, adenovirus; LV, lentivirus. Reproduced in whole from Ref (Allemailem et al., 2023). (Open Access – Licensee: Dovepress, under Creative Commons (CC-BY) license.).

Recent advancements in the realm of CRISPR/Cas9 technology have fundamentally transformed genome editing, altering its landscape across cellular differentiation and immune response modulation. This breakthrough has shed light on cancer progression mechanisms, paving the way for potential breakthroughs in antitumor immunotherapy (Shimizu et al., 2023). Utilizing CRISPR/Cas9, researchers now engineer universal T-cells armed with either recombinant T-cell receptor (TCR) or chimeric antigen receptor (CAR), while also leveraging its capabilities in cytokine stimulation, antibody design, natural killer (NK) cell transfer, and circumventing immune checkpoints. This innovation has significantly contributed to adoptive cell transfer immunotherapy, with some approaches gaining FDA approval. By manipulating immunogenetic regulators, CRISPR/Cas9 has provided a novel framework for immuno-oncology screening. Previously deemed unattainable, this strategy has demonstrated efficacy in treating various cancers such as lymphoma, melanoma, lung, and liver malignancies. However, the efficient and safe delivery of CRISPR/Cas9 into immune cells remains a formidable challenge, necessitating the exploration of diverse targeting methodologies including virus-mediated, electroporation, microinjection, and nanoformulation-based techniques, each presenting its own set of constraints. Excellent review updates in cancer management through the marriage of immunotherapy and CRISPR/Cas9 technology, exploring innovative approaches for targeting this genome-editing system within immune cells as a novel anticancer strategy (Wu and Cao, 2019; Ghaffari et al., 2021; Allemailem et al., 2022; Allemailem et al., 2023). Overall, advanced gene editing technologies, such as CRISPR/Cas9, allow precise modification of APCs' genome to enhance their immunomodulatory functions. This includes knockout of pro-inflammatory genes or insertion of therapeutic genes for targeted immune regulation. There is a definite need for improved delivery approaches and expression of CRISPR/Cas9 system *in vivo* (Figure 2).

### 4.2 Nanoparticle delivery systems

This section offers a succinct overview – far from being exhaustive – of nanoparticle-based delivery systems for immunomodulatory agents. As such, the section also aims to provide a concise introduction to the topic, given that we have extensively covered the complexities and nuances of nanoparticle-based delivery systems in our previous work (Puccetti et al., 2023a; Puccetti et al., 2023b; Puccetti et al., 2024).

The detailed discussion on the materials used in these systems, including polymers, lipids, metals, and hybrid materials, as well as their respective advantages and limitations, is elaborated upon in our earlier publications. We have also examined various nanoformulations that have shown promise, such as liposomes, polymeric nanoparticles, and dendrimers, highlighting specific instances where these formulations have effectively enhanced the delivery and efficacy of immunomodulatory agents (Puccetti et al., 2023a; Puccetti et al., 2023b; Puccetti et al., 2024).

Moreover, our comprehensive analyses have addressed why nanodrug delivery systems are pivotal. These systems offer targeted delivery, improved bioavailability, and controlled release, which are essential for enhancing therapeutic outcomes and minimizing side effects. By providing a more targeted and controlled approach, nanoparticle-based delivery systems can significantly improve the precision and effectiveness of immunomodulatory therapies.

Thus, while this section only recapitulates major points of interest, readers seeking more detailed information are encouraged to refer to our extensive prior work, where we have thoroughly explored the materials, successful nanoformulations, and the rationale behind using nanodrug delivery systems. This foundational understanding is crucial for appreciating the broader implications and future potential of nanoparticle-based immunomodulation. Those points include:

#### 4.2.1 Encapsulation of immunomodulatory agents

Nanoparticles can be engineered to encapsulate immunomodulatory agents, such as small molecules, peptides, nucleic acids, or biologics. These NPs can then be targeted to APCs, either via surface modifications or passive uptake mechanisms, to deliver the therapeutic payload directly to the immune cells (Puccetti et al., 2023a; Puccetti et al., 2023b; Jeong et al., 2023; Puccetti et al., 2024).

#### 4.2.2 Controlled release kinetics

Nanoparticle delivery systems offer control over the release kinetics of immunomodulatory agents, ensuring sustained and localized delivery to APCs within the diseased microenvironment. This spatiotemporal control enhances therapeutic efficacy while minimizing systemic toxicity. Innovative strategies for delivering cancer immunotherapy in a safer and more controlled fashion could broaden the therapeutic reach to more patients and decrease toxic side effects. Enhanced delivery methods, in particular, can increase the concentration of immunotherapies in affected tissues, improve targeting of specific tumors or immune cells, and minimize off-target adverse reactions. Ongoing research aims to create new delivery systems for immunotherapies, including, implants, scaffolds, biomaterials, and cell-based platforms. Various materials, such as lipids, polymers, and metals, have been employed to develop these technologies, and we refer readers to existing literature for detailed discussions on these materials. Delivery systems offer numerous advantages over standalone therapeutic agents. This article explores how these platforms can be utilized for more effective and safer cancer immunotherapy. Firstly, they can be designed to protect therapeutic agents until they reach the target cells. Secondly, delivery systems can provide spatiotemporal control over therapeutics, activating them only in response to specific stimuli like pH, light, or ultrasound, ensuring the cargo remains inactive until it reaches the target cells. Lastly, delivery platforms such as implants enable localized, controlled drug release, and cell therapies have been developed to reduce the toxicities linked with systemic administration (Riley et al., 2019; Han et al., 2021; Pérez-Herrero et al., 2024).

#### 4.2.3 Enhanced cellular uptake

Surface functionalization of nanoparticles with ligands targeting APC-specific receptors, such as mannose receptors or scavenger receptors, facilitates efficient cellular uptake and internalization by APCs. This targeted delivery approach enhances the specificity and potency of immunomodulatory interventions. To further enhance the potential of NPs in these areas, a deeper understanding of the regulation of adaptive immune responses at high mechanistic and functional levels has revealed numerous strategies to modulate them for specific outcomes, marking the advent of immunotherapies. It is now evident that to achieve a significant therapeutic effect, designed formulations must target specific compartments of the immune system. Current immunotherapy efforts focus on developing specific effector cells, particularly B and T lymphocytes. One of the most promising immunotherapies today is immune checkpoint blockade (ICB), which counters processes such as T cell exhaustion by targeting inhibitory molecules. Discovering the roles of molecules like Cytotoxic T lymphocyte antigen 4 (CTLA-4) and Programmed cell death 1 (PD-1) led to the development of monoclonal antibodies that interfere with these markers within the immune synapse, restoring the functionality of immune cells. The FDA has approved the use of ICB monoclonal antibodies against CTLA-4 (ipilimumab) and PD-1 (pembrolizumab and nivolumab). B and T lymphocytes, particularly the latter, which this review focuses on, are the primary effector cells of the adaptive immune system. Therefore, developing pathogen-specific B and T cell immunity is the primary goal in the pursuit of both therapeutic and prophylactic interventions (Moser et al., 2017; Chen et al., 2021; Rennick et al., 2021; Singh et al., 2021; Perez-Potti et al., 2023).

**FIGURE 3:**
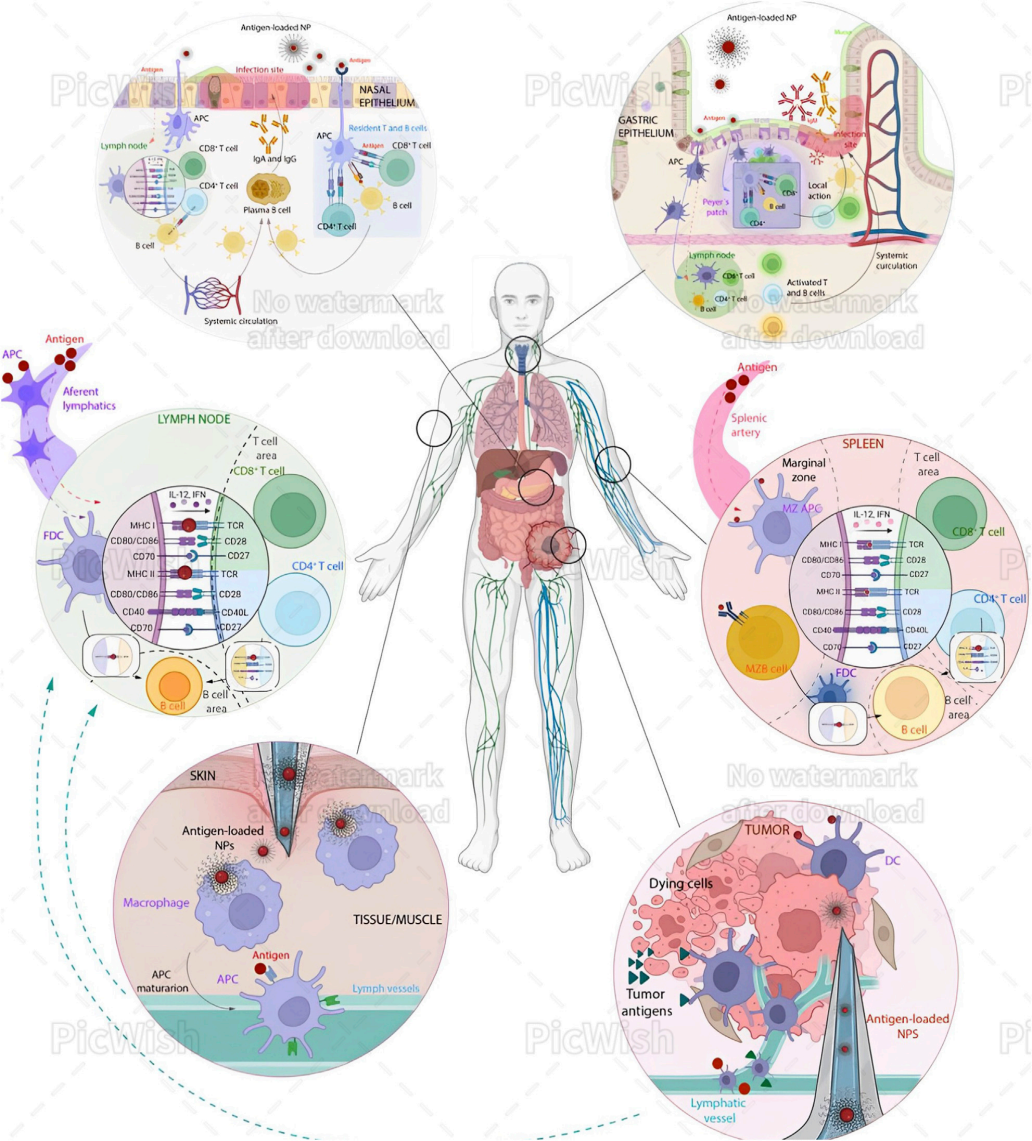
Immune effects of nanoparticles (NPs) following different administration routes. Intratracheal (IN) Administration: Microformulations (Brown et al., 2023; Puccetti et al., 2023c) and NPs are taken up by antigen-presenting cells (APCs), which process the antigens and may occasionally passively diffuse to lymph nodes (LNs). The processed antigens are then transported to the proximal LN, where they are presented to CD8^+^ T cells, CD4^+^ T cells, and B cells. These activated immune cells then concentrate at the infection site in the nasal epithelium, exerting cytotoxic activity and promoting IgA secretion. Additionally, APCs can present antigens directly to tissue-resident T cells, eliciting a rapid local response. Oral Administration: NPs must navigate the acidic pH and mucus of the gastric epithelium. APCs, often M cells, internalize the NPs. Once processed, antigens are presented either in the mesenteric lymph node or directly in the Peyer’s patches, activating CD4^+^ T cells, CD8^+^ T cells, and B cells. This activation leads to local production of IgA and IgM at the infection site. Intravenous (IV) Administration: NPs reach the spleen via the splenic artery. APCs in the marginal zone internalize and process the antigens. These APCs then migrate to the T cell area, presenting antigens to CD4^+^ and CD8^+^ T cells. Additionally, B cells from the marginal zone can internalize antigens and present them to follicular dendritic cells (FDCs). Subsequently, CD4^+^ T cells and FDCs present the antigens to immature B cells in the B cell region, triggering an immune response. Intramuscular/Subcutaneous (IM/SC) Administration: This is a common immunization strategy where NPs are injected under the skin or into muscular tissues, where macrophages take them up. These macrophages mature into APCs and migrate to LNs via the afferent lymphatic vessels. Occasionally, antigens can also diffuse through these vessels to the LNs. There, FDCs present the antigens to B and T cells, activating CD8^+^ and CD4^+^ T cells through MHC I and II signaling over T cell receptors. Intratumoral (IT) Administration: This approach has gained attention for cancer treatment. NPs are processed similarly to the IM/SC route but can enhance the antitumor immune response by triggering immunogenic cell death (ICD) and providing new antigens for DCs. This process activates cytotoxic infiltrating T cells in the tumor. Reproduced in whole from Ref (Perez-Potti et al., 2023). (Open Access, under CC BY-NC-ND 4.0 DEED; Licensee, ELSEVIER.)

Figure 3 illustrates the variety of immune effects of nanoparticles following differential administration routes.

Undoubtedly, the administration route significantly influences how NPs interact with the immune system (Figure 3). Understanding these interactions is vital for designing nanoparticles for therapeutic and diagnostic applications, ensuring they elicit the desired immune response while minimizing adverse effects. This area of research continues to evolve, with ongoing studies aimed at optimizing the use of NPs in medicine. In general terms, the major areas of interest include—NP properties: Size, shape, and surface modifications influence immune interactions; and safety: Ensuring biocompatibility and minimizing toxicity are crucial for medical applications. Understanding these differences is essential for designing safe and effective nanoparticle-based therapies. As an example, upon intravenous (IV) administration of NPs, the immediate exposure may result in NPs facing immediate immune recognition and clearance by the liver and spleen. “Stealth coating” of NPs with materials like PEG can reduce immune detection and prolong circulation (Puccetti et al., 2023a). Thus different administration routes of nanoparticles (NPs) trigger distinct immune responses—(*i*) Intratracheal (IN): NPs are taken up by APCs, processed, and presented to T and B cells in lymph nodes, leading to local immune responses in the nasal epithelium; (*ii*) Oral: NPs navigate the gastrointestinal tract, are internalized by APCs, and activate T and B cells in mesenteric lymph nodes or Peyer’s patches, producing local IgA and IgM; (*iii*) Intravenous (IV): NPs reach the spleen, where APCs present antigens to T and B cells, inducing a systemic immune response; (*iv*) Intramuscular/Subcutaneous (IM/SC): NPs are taken up by macrophages at the injection site, which migrate to lymph nodes, activating T and B cells; (*v*) Intratumoral (IT): NPs enhance antitumor immune responses by triggering immunogenic cell death and activating cytotoxic T cells in the tumor.

Nanomedicine and the use of NPs may still be in their infancies. Notwithstanding these limitations, nanomedicine, in general, offers significant advancements in human disease therapy by providing targeted, cell-specific treatments, controlled drug release, and personalized dosages. This innovative approach enhances treatment efficacy and minimizes side effects by using stimuli-responsive nanoparticles to deliver drugs precisely to affected tissues. It holds particular promise for cancer, genetic, and chronic diseases. Additionally, personalized treatment plans and wearable drug delivery devices improve patient compliance and therapeutic outcomes. Companion diagnostics further optimize treatment by allowing real-time monitoring and adjustments. Overall, nanomedicine represents a revolutionary step forward in improving patient care and treatment success (Puccetti et al., 2024).

### 4.3 *Ex Vivo* manipulation techniques: these include

#### 4.3.1 Isolation and expansion of APCs

*Ex vivo* manipulation involves isolating APCs, such as dendritic cells or macrophages, from patient-derived samples and expanding them in culture (Jonny et al., 2024). These expanded APC populations can then be genetically modified or loaded with immunomodulatory agents before reinfusion into the patient. Recent reviews deal exhaustively with this issue, mostly in regards to the use of DCs as immunogenic vaccines in cancer immunotherapy, as a promising approach to address limitations encountered by immune checkpoint blockades and adoptive cell transfer therapies. One such review (Lee et al., 2023) explores the evolution from classical DC vaccines to new generations, such as biomaterial-based, immunogenic cell death-inducing, mRNA-pulsed, DC small extracellular vesicle (sEV)-based, and tumor sEV-based DC vaccines. These innovative DC vaccines aim to enhance antitumor immune responses and overcome challenges faced by conventional approaches. Classical DC vaccines involve *ex vivo* differentiation of DCs and direct targeting of antigens to DCs *in vivo.* However, their efficacy is limited due to the immunosuppressive tumor microenvironment and inadequate T cell priming and activation. This highlights the importance of new generations of DC vaccines that can better stimulate antigen-specific immune responses, promote T cell activation and memory, and overcome immunosuppressive factors in the tumor microenvironment. Specific strategies discussed include biomaterial-based DC vaccines that recruit and activate endogenous DCs *in situ*, and combinatory approaches with immunogenic cell death-inducing therapies like radiotherapy, photodynamic therapy (PDT), and photothermal therapy (PTT) to enhance antitumor immune responses. These approaches aim to convert cold tumors into hot tumors by inducing immunogenic cell death, promoting antigenicity, and activating DCs to elicit robust antitumor immunity. Overall, these considerations emphasize the potential of new generation DC vaccines to revolutionize cancer immunotherapy by enhancing DC function, improving T cell responses, and overcoming challenges encountered by traditional approaches in the context of cancer treatment (Lee et al., 2023).

#### 4.3.2 Stimulation, maturation, and phenotype switching protocols

*Ex vivo* manipulation allows for the stimulation and maturation of APCs under defined culture conditions, mimicking the physiological cues encountered within the diseased microenvironment. This process primes APCs for enhanced antigen presentation and immunomodulatory functions upon reinfusion. Future directions, including optimization of engineering techniques, identification of novel target antigens, and exploration of combinatorial treatment approaches have been extensively reviewed elsewhere (Han et al., 2019; Que et al., 2020).

As mentioned earlier, a recent article discusses the activation of the tryptophan metabolic enzyme IDO1 in conventional dendritic cells (cDCs) and its role in maintaining tolerance and regulating immune responses (Gargaro et al., 2022). Specifically, it focuses on the interaction between different subsets of cDCs, namely cDC1 and cDC2, in promoting a tolerogenic environment through metabolic communication. It was found that: (*i*) the IDO1 pathway is expressed in mature cDC1 but not in cDC2; (*ii*) Mature IDO1^+^ cDC1 exhibit regulatory functions both *in vitro* and *in vivo*; (*iii*) The IDO1-competent cDC1 induce regulatory cDC2 through metabolic communication involving tryptophan metabolism; (*iv*) l-kynurenine, a tryptophan metabolite, plays a role in recruiting AhR-competent cDC2 into a tolerogenic pool. Thus, the study identifies a metabolic axis where IDO1-expressing cDC1 cells extend their regulatory capacity to cDC2 through the production of l-kynurenine. The article also delves into the mechanisms involved in IDO1 expression in cDC subsets, the involvement of transcription factors like IRF8 and Batf3, as well as the impact of IL-6 in regulating IDO1 expression in cDCs. Additionally, it explores the functional implications of IDO1 in the context of autoimmune diseases like experimental autoimmune encephalomyelitis (EAE). These findings suggest that cDC1 play a crucial role in maintaining immune tolerance through the IDO1 pathway and metabolic communication with cDC2. In line with several previous studies, this study highlights the potential therapeutic implications of targeting this metabolic axis in autoimmune demyelinating diseases (Grohmann and Puccetti, 2015; Mondanelli et al., 2017; Mondanelli et al., 2020; Zelante et al., 2024).

Specular to this perspective – but relevant to targeted drug delivery strategies in IDO1-related contexts (i.e., cancer immunotherapy) – is a recent report illustrating how reprogrammed IDO-induced immunosuppressive microenvironment synergizes with immunogenic magnetothermodynamics for improved cancer therapy. The Authors developed a magnetic vortex nanodelivery system for the targeted delivery of the IDO inhibitor NLG919, integrated with magnetic hyperthermia therapy to reverse the immune-suppressive microenvironment of liver cancer and inhibit tumor growth. This system comprises thermoresponsive polyethylenimine-coated ferrimagnetic vortex-domain iron oxide nanorings (PI-FVIOs) loaded with NLG919 (NLG919/PI-FVIOs). Under thermal effects, NLG919 can be precisely released from the delivery system, counteracting IDO-mediated immune suppression and synergizing with NLG919/PI-FVIOs-mediated magnetothermodynamic therapy-induced immunogenic cell death, resulting in effective hepatocellular carcinoma suppression. *In vivo* studies demonstrated that this combination therapy significantly inhibits tumor growth and metastasis by enhancing the accumulation of cytotoxic T lymphocytes and suppressing regulatory T cells within the tumor. Overall, the Authors’ findings reveal that NLG919/PI-FVIOs can induce a potent antitumor immune response by disrupting the IDO pathway and activating immunogenic cell death, offering a promising therapeutic avenue for hepatocellular carcinoma treatment (Wang et al., 2024).

Traditional cancer immunotherapies, such as T cell-based treatments, have limited success due to the immunosuppressive tumor microenvironment dominated by tumor-associated macrophages (TAMs). TAMs generally support tumor growth and resistance to treatment, but some can stimulate an immune response. Targeting TAMs has been difficult due to their high plasticity and the lack of specific immunological direction. A recent study investigated the potential for myeloid cell-based cancer-immunotherapy methods to induce TAMs to produce interleukin-12 (IL-12), an anti-tumor cytokine, using a combination of small molecules and nanoparticle delivery systems. The study combined a high-throughput molecular screen for IL-12-inducing compounds with cyclodextrin-based nanoparticles to deliver these compounds effectively to macrophages. The novel nanoparticle – encapsulating three small-molecule drugs targeting the JAK1/2, NF- κB, and TLR pathways – was successful in inducing IL-12 production in TAMs. RNA sequencing was performed on stimulated bone marrow-derived macrophages, which displayed a novel TAM phenotype characterized by an over-expression of IL-12, MARCO, DC-SIGN, and SIGNR7 and the absence of interferon-stimulated genes, including those encoding the inhibitory factors PDL1 and IDO1/2 (Ge, 2024).

#### 4.3.3 Integration with cell-based therapies via merging advanced technology with innovative drug delivery systems

Engineered APCs can be integrated into cell-based immunotherapies, such as adoptive cell transfer or chimeric antigen receptor (CAR) T cell therapy. By modulating APC function *ex vivo*, these cell-based therapies harness the innate antigen-presenting capabilities of APCs to enhance therapeutic outcomes. Significant advancements in drug delivery technologies enabled by micro- and nanotechnologies at the intersection of engineering, science, and medicine. These technologies, such as microelectromechanical systems (MEMS), allow for the development of novel drug delivery devices ranging from microparticles to implantable pumps. The recent literature highlights the rapid evolution of drug delivery methods over time, from conventional formulations to the recently approved “digital medicines” incorporating ingestible microelectronic sensors. Several companies have made significant advancements in this field, such as MicroCHIPS and *Proteus* Digital Health, with their respective wireless drug delivery devices and ingestible sensors. There is increased recognition of the potential of these technologies to transform medicine by enabling precise drug delivery and improving patient compliance. The issue of integration with cell-based therapies – and fully merging advanced technology with innovative drug delivery systems as well – requires combining knowledge from international experts in various disciplines, exploring novel techniques grounded in engineering principles to achieve diverse drug release profiles (Puccetti et al., 2024). Microneedles, oral drug delivery platforms, and self-folding container technologies are discussed as innovative approaches to drug delivery. The use of micro- and nanotechnologies aims to enhance drug efficacy, reduce invasiveness, and tailor drug release profiles to meet clinical needs. However, there occur challenges in translating these technologies from the laboratory to clinical use and this underscores the importance of continued research and development. Various pumping systems, including osmotic pumps and electronically operated platforms, are reviewed for transdermal and subcutaneous drug delivery. Additional issues involve implantable pumping strategies for diverse applications, such as intraocular drug delivery and cancer therapies, as well as the challenges of drug delivery to delicate structures like the inner ear. Overall, the bulk of the data highlights the promising opportunities presented by micro- and nanotechnologies in drug delivery and emphasizes the need for ongoing investment and development to bring these technologies to the market (Isser et al., 2021; Lee et al., 2023; Niu et al., 2023; Yang et al., 2023; Pandit et al., 2024).

Some of the foundational concepts underlying the largely speculative theoretical bases discussed thus far are summarized, in general terms, in Box 2.

BOX 2 **Engineered antigen-presenting cells (APCs).**

APCs play a crucial role in the immune system by presenting antigens to T cells, initiating and regulating immune responses. In autoimmune diseases, the presentation of self-antigens leads to an aberrant immune response. By engineering APCs to present antigens in a way that induces tolerance rather than activation, researchers aim to re-educate the immune system to recognize these antigens as non-threatening.

•**Means of Merging Advanced Technologies:** Engineered APCs are modified to express self-antigens in the context of immunoregulatory signals that promote tolerance. This can be achieved by altering the expression of co-stimulatory and inhibitory molecules on the APC surface or by delivering immunomodulatory cytokines alongside the antigen presentation (Grohmann et al., 2007).
•**Advantages:** This method offers a highly specific approach to treating autoimmunity, potentially reducing the need for broad immunosuppression and minimizing side effects. By directly targeting the pathogenic immune response, engineered APCs can provide more sustained and effective disease control.
Potential Routes of Administration
  • **Intravenous (IV) Administration:** Direct infusion of engineered APCs into the bloodstream allows for systemic distribution and interaction with immune cells throughout the body. This route is particularly useful for systemic autoimmune diseases like lupus or rheumatoid arthritis.
  • **Intratumoral or Local Injection:** For localized autoimmune conditions, such as type 1 diabetes (targeting pancreatic islets) or multiple sclerosis (targeting CNS lesions), local administration can concentrate the therapeutic effect where it is most needed.
  • **Subcutaneous Administration:** This less invasive route allows for slower, sustained release of engineered APCs, which can be beneficial for maintaining long-term tolerance induction.
Expected Benefits
  • **Specificity and Precision:** Engineered APC therapies can be tailored to target specific autoantigens involved in the disease process, reducing off-target effects and improving efficacy.
  • **Reduced Side Effects:** By focusing on tolerance induction rather than broad immunosuppression, these therapies minimize the risk of infections and other complications associated with generalized immune suppression.
  • **Long-term Disease Control:** Inducing immune tolerance has the potential to provide lasting remission, reducing or eliminating the need for continuous treatment.
  • **Personalization:** APCs can be customized for individual patients based on their unique antigenic profiles, enhancing the effectiveness of the therapy.

In summary, the development of engineered APCs for tolerance induction represents a cutting-edge approach in the treatment of autoimmune diseases. By leveraging the precise modulation of the immune system, these cell therapies offer the potential for highly specific, effective, and long-lasting disease control, heralding a new era in autoimmune disease management.

## 5 Clinical trials

Multiple experimental and clinical studies investigating *ex vivo*-generated DC immunotherapy for autoimmune diseases have recently been summarized in Ref. (Jonny et al., 2024). In rheumatoid arthritis patients, administering DC immunotherapy intraarticularly with autologous synovial fluid has shown good safety, tolerability, and efficacy, as indicated by the absence of flares and increased disease severity during the observation period (Bell et al., 2017). Phase I trials in type 1 diabetes mellitus (T1DM) subjects indicated that DC immunotherapy was well-tolerated with no serious adverse events, although no clinical improvements were observed (Giannoukakis et al., 2011). Another study on T1DM subjects found that DC immunotherapy with pancreatic islet cell antigens was safe and well-tolerated, inducing immune tolerance for up to 3 years post-therapy. This study also noted a temporary decrease in CD4^+^ and CD8^+^ T cell responses to pancreatic islet cell autoantigens, along with an increase in regulatory and memory CD4^+^ T cells after the initial injection (Nikolic et al., 2022).

In particular, tolerogenic dendritic cells are being extensively investigated as a promising therapy, either alone or in combination with other treatments, for T1DM. While tolerogenic DCs have been tested in human trials for various autoimmune diseases, T1DM presents unique challenges. Notably, the NOD mouse model, commonly used in T1DM research, naturally develops diabetes, unlike many disease-induced animal models of autoimmune diseases. Evidence has accumulated from animal studies, particularly using NOD mice, on different tolerogenic DC approaches. Key aspects of this cell-based therapy include tolerogenic DC preparation protocols, antigen-specific versus nonspecific methods, dosing, application schemes and routes, tolerogenic DC migration, and their effects in prevention, early intervention, or treatment. However, the bulk of the data on tolerogenic DC therapy in preclinical research indicates that further investigation is imperative for effective tolerogenic DC treatments for T1DM in humans (Funda et al., 2019).

Phase IB trials of DC immunotherapy in multiple sclerosis patients showed no disease worsening during follow-up, along with an increase in IL-10 and regulatory T cells (Zubizarreta et al., 2019). In those trials, cell-based therapy involved intra-articular (Bell et al., 2017), intradermal (Horwitz, 2008; Giannoukakis et al., 2011) or intravenous (Zubizarreta et al., 2019) administrations. The clinical trials discussed demonstrate that DC immunotherapy can be performed with reasonable safety and potential effectiveness in autoimmune patients. However, neither for CAR-T cell nor for engineered DC-based therapy in autoimmunity, clinical trials have not so far gone beyond Phase 1 or 2. In this regard, Ref (Blache et al., 2023) provides a summary of clinical trials using different CAR-T cell types for the treatment of various autoimmune diseases. Therapies targeting pathogenic T cells have been shown to alter the disease course and preserve β-cell mass only in the short term, providing evidence that restoring the balance between pathogenic T cells and Tregs is not sufficient to cure T1D. To this end, regulatory cell-based approaches, either Tregs or tolerogenic DCs, have been proposed for a definitive therapy for T1D patients (Warshauer et al., 2020).

Multiple sclerosis is an autoinflammatory condition that damages myelinated neurons. Disease-modifying treatments slow relapsing-remitting disease, but most patients progress to secondary progressive disease, which remains largely unresponsive, and there is no effective treatment for primary progressive MS. Innate and adaptive immune cells in the CNS are crucial in initiating autoimmune attacks and maintaining chronic inflammation. This review focuses on the role of regulatory T cells in suppressing MS progression and promoting remyelination and repair of CNS lesions. The potential to genetically reprogram regulatory T cells to achieve localized immune suppression and repair while maintaining a competent immune system is discussed. Future reprogrammed regulatory T cells could offer lasting disease suppression after a single treatment cycle (Zhong and Stauss, 2024). At the time of this writing, the successful targeting of malignant B cells using CAR-T cell therapy has sparked interest in its potential application for eliminating pathogenic B cells in autoimmune diseases. Early findings indicate possible effectiveness, but the research is still in its infancy with small sample sizes, a lack of controlled trials, and an unclear role of immunodepletion. Additionally, the optimal CAR-T constructs and the most suitable patient groups for this treatment have yet to be determined (Daamen and Lipsky, 2024).

Overall, cell-based immunotherapy does hold promise for autoimmunity treatment, though further research is needed (Shumnalieva et al., 2024). On a positive note (Blache et al., 2023), there are two registered clinical trials involving CAR Tregs aimed at inducing immunological tolerance, but these are focused on a different context—solid organ transplantation (Henschel et al., 2023). In these trials, the strategy involves directing Tregs via a CAR receptor to target human leukocyte antigen (HLA) molecules present on the transplanted organ, thereby promoting immunotolerance and preventing organ rejection (Proics et al., 2023). Specifically, these clinical trials involve kidney and liver transplantation and utilize CAR Tregs that target HLA-A2.

## 6 Ex vivo-generated tolerogenic dendritic cells: challenges and hope for an effective therapy of autoimmune diseases

Understanding immune mechanisms from autoimmune diseases has propelled the development of various strategies to generate tolerogenic dendritic cells, which are extensively reviewed in Ref (Jonny et al., 2024). Such DCs are anticipated to provide long-term suppression of unwanted immune responses and restore systemic immune tolerance. Notably, autologous dendritic cell transfers have shown high tolerability without significant side effects, suggesting their potential as a long-term therapy for autoimmune conditions.

Several methods have been devised to induce the tolerogenic phenotype in DCs *ex vivo*. Tolerogenic DCs can be generated by culturing DCs with immunosuppressive agents, anti-inflammatory cytokines, or probiotics. Alternatively, genetic manipulation using viral vectors to express immunosuppressive phenotypes, such as CTLA-4 and IDO genes, is another approach. Despite the various methods available to create tolerogenic DCs, it is crucial to compare their efficacy in promoting tolerance in autoimmune diseases, particularly in systemic lupus erythematosus (SLE). In SLE, the chronic inflammatory environment may alter the tolerogenic DC phenotype to become autoreactive post-transfer. Therefore, ensuring tolerogenic DCs maintain their tolerogenic phenotype under inflammatory conditions is essential for controlling autoimmunity in SLE. Overall, tolerogenic DCs generated via various protocols exhibit common features: a semi-mature phenotype, resistance to maturation, T-cell hypo-responsiveness, secretion of immunosuppressive cytokines, and promotion of Treg induction (Müller et al., 2024).

Dendritic cells can induce specific tolerance to antigens, and tolerogenic DCs presenting autoantigens are expected to enhance central and peripheral tolerance. However, identifying specific antigens for systemic tolerance induction remains challenging. In organ-specific autoimmune diseases, parts of the affected organ can serve as sources of autoantigens, as seen in clinical trials using synovial fluid for DC therapy in rheumatoid arthritis. Conversely, in systemic autoimmune diseases like SLE, using a single organ’s antigens may not represent the diverse autoantigens involved (Nunez et al., 2023).

SLE is driven by impaired clearance of apoptotic or necrotic cells, where self-DNA, RNA, histones, and nucleosomes trigger autoimmune responses. Preclinical studies using histone proteins have shown promising results in symptom reduction, though they did not demonstrate tolerance induction via Treg formation or anergy. Hence, designing specific antigens to induce tolerance in SLE remains a significant challenge (Nunez et al., 2023).

Evidence suggests that tolerogenic DCs exposed to specific antigens (loaded tolerogenic DCs) might be less effective in inducing tolerance compared to those not exposed to antigens (unloaded tolerogenic DCs). Unloaded tolerogenic DCs could be more effective in systemic autoimmune diseases like SLE, where autoantigens are persistently present. Research in T1DM mouse models has shown that unloaded t tolerogenic DCs can promote antigen-independent regulatory T cell expansion. However, unloaded tolerogenic DCs have an unstable phenotype and may become immunogenic upon antigen exposure in the body, potentially worsening autoimmunity. Therefore, it is crucial to study the phenotypic and functional changes in unloaded tolerogenic DCs after their transfer into autoimmune patients and to develop methods to maintain their tolerogenic properties.

In clinical settings, immunosuppressant agents for SLE can have adverse side effects. However, preclinical studies in SLE models suggest that DC therapy can initiate immune tolerance without significant side effects, indicating its superiority over standard treatments. The administration route of DCs requires careful consideration. Although intraarticular tolerogenic DCs administration in rheumatoid arthritis has shown symptom reduction, its invasiveness poses challenges for repeated dosing.

Given the relapsing nature of autoimmune diseases, tolerogenic DCs therapy may require repeated dosing. Thus, the administration route should be non-invasive and allow effective migration to lymphoid organs to induce systemic immune tolerance. Although a definitive approach to fully restore immune tolerance has not been found, autologous DC transfer could potentially reduce reliance on immunosuppressant agents in patients with autoimmune disease.

## 7 Further elaboration on future directions for APC-based immunotherapy

While this review provides an overview of the future directions for antigen-presenting cell (APC)-based immunotherapy, there may be a need for more detailed, actionable insights to guide researchers. Box 3 aims to elaborate on the recommendations, specifically addressing the technical challenges associated with APC engineering and proposing innovative solutions to overcome these hurdles.

BOX 3 **| Technical Challenges in APC Engineering.**

1. Efficient APC Isolation and Expansion:
  - Challenge: Isolating APCs in sufficient quantities and expanding them *ex vivo* without losing their functional properties – or “immature” condition – is a significant technical challenge.
  - Innovative Approach: Development of advanced culture systems that mimic the natural microenvironment of APCs can help maintain their phenotype and function during expansion. Utilizing bioreactors and 3D culture systems can enhance the yield and quality of APCs.
2. Genetic Modification of APCs:
  - Challenge: Introducing genetic modifications to APCs to enhance their immunoregulatory capacity (e.g., fostering Treg generation) or to express specific antigens is technically demanding. Ensuring stable and efficient gene integration without affecting cell viability and function is crucial.
  - Innovative Approach: CRISPR/Cas9 technology and other genome editing tools offer precise methods for genetic modification. Developing non-viral delivery systems, such as nanoparticle-based methods, can improve the safety and efficiency of gene editing in APCs.
3. Optimization of Antigen Loading:
  - Challenge: Efficiently loading APCs with antigens to ensure appropriate T-cell subset activation is a complex process. The method of antigen delivery and processing within APCs can significantly impact the outcome.
  - Innovative Approach: Exploring various antigen delivery systems, such as liposomes, dendrimers, and cell-penetrating peptides, can enhance antigen uptake and presentation. Additionally, using synthetic biology approaches to engineer APCs with optimized antigen processing machinery can improve their efficacy.
4. Enhancing APC Migration and Homing:
  - Challenge: Ensuring that engineered APCs can migrate to and home in on the appropriate lymphoid tissues or tumor microenvironments is vital for effective immunotherapy.
  - Innovative Approach: Engineering APCs to express specific chemokine receptors or adhesion molecules can improve their migration and homing capabilities. Utilizing *in vivo* imaging techniques can also aid in tracking and optimizing APC distribution.
5. Overcoming Immunosuppressive Environments:
  - Challenge: APCs often face immunosuppressive environments when dealing with tumor immunotherapy, which can hinder their function and survival.
  - Innovative Approach: Combining APC-based therapies with checkpoint inhibitors or other immunomodulatory agents can help counteract immunosuppression by the tumor microenvironment. Engineering APCs to secrete cytokines or other factors that modulate the tumor microenvironment can also enhance their therapeutic potential.
Proposed Innovative Approaches:
  1. Synthetic APCs:
    - Developing synthetic APCs using biomaterials and nanotechnology to mimic the natural properties of APCs. These synthetic constructs can be designed to present multiple antigens and provide co-stimulatory/co-inhibitory signals, potentially offering a more controlled and reproducible approach.
  2. Multi-Omics Integration:
    - Utilizing multi-omics technologies (genomics, proteomics, metabolomics) to gain a comprehensive understanding of APC biology. This can inform the rational design of APC-based therapies and identify key pathways for targeted manipulation.
  3. Machine Learning and Computational Modeling:
    - Applying machine learning algorithms and computational models to predict the behavior of engineered APCs and optimize their design. These tools can help identify the most effective genetic modifications and culture conditions.
  4. Clinical Translation and Standardization:
    - Developing standardized protocols for APC engineering and clinical-grade manufacturing to facilitate the translation of research findings into clinical applications. Establishing robust quality control measures is essential for ensuring the safety and efficacy of APC-based therapies.

## 8 Conclusion

Biotherapies for autoimmunity include monoclonal antibodies that target specific antigens like TNF-α and IL-6, fusion proteins that inhibit immune pathways such as CD80/CD86-CD28, cytokine modulators that block cytokines like IL-1 and IL-17, JAK inhibitors that interfere with cytokine signaling, and cell-based therapies like stem-cell transplantation and regulatory T cell therapy. These treatments aim to modulate the immune system precisely, offering targeted interventions to reduce pathological immune responses and improve patient outcomes.

Biotherapies offer significant benefits in treating autoimmune diseases, providing targeted action with fewer side effects and improving patient outcomes and quality of life. However, challenges include high costs, potential side effects like infections, and the development of resistance or loss of response over time. Long-term safety remains a concern, necessitating ongoing research and monitoring. Despite these challenges, biotherapies have transformed the treatment landscape, offering more personalized and effective options for managing autoimmune conditions.

One of the most promising future directions in biotherapy for autoimmunity involves cell therapies using engineered APCs to induce immune tolerance. This approach focuses on reprogramming the immune system to tolerate self-antigens, thereby preventing or reducing autoimmune attacks.

Intravenous administration might exploit direct infusion of engineered APCs into the bloodstream allows for systemic distribution and interaction with immune cells throughout the body. This route is particularly useful for systemic autoimmune diseases such as lupus or rheumatoid arthritis. For localized autoimmune conditions, such as type 1 diabetes (targeting pancreatic islets) or multiple sclerosis (targeting CNS lesions), local administration can concentrate the therapeutic effect where it is most needed. Subcutaneous administration my be less invasive route allows for slower, sustained release of engineered APCs, which can be beneficial for maintaining long-term tolerance induction (Fallarino et al., 2009; Luca et al., 2010; Arato et al., 2020).

Expected benefits would include specificity and precision: Engineered APC therapies can be tailored to target specific autoantigens involved in the disease process, reducing off-target effects and improving efficacy. Reduced side effects could be achieved by focusing on tolerance induction rather than broad immunosuppression, these therapies minimize the risk of infections and other complications associated with generalized immune suppression.

An additional benefit may consist of long-term disease control: Inducing immune tolerance has the potential to provide lasting remission, reducing or eliminating the need for continuous treatment. Moreover, personalization could be improved: APCs could be customized for individual patients based on their unique antigenic profiles, enhancing the effectiveness of the therapy.

Overall, tolerogenic DCs present a promising approach for treating autoimmune disorders by re-educating and modulating immune responses in an antigen-specific way, thereby minimizing side effects on the immune system compared to standard immunosuppressive therapies. Research has demonstrated the safety and efficacy of DC therapy in various experimental models of autoimmune diseases, including multiple sclerosis, T1D, and rheumatoid arthritis. Additionally, phase I clinical trials have indicated that DC therapy is safe and well-tolerated in patients with MS, T1D, and rheumatoid arthritis. However, optimizing several parameters is necessary to enhance DC efficacy. One crucial aspect to determine is the optimal route of administration. Various delivery methods, such as intravenous, subcutaneous, intraperitoneal, intradermal, intranodal, and intraarticular routes, have been explored in experimental models and phase I clinical trials.

By employing these diverse engineering strategies, researchers have been aiming to harness the immunomodulatory potential of DCs for the precise delivery of therapeutic biologicals in pathogenic immunity. Each approach offers unique advantages and challenges, highlighting the need for continued innovation and optimization in the field of APC-based immunotherapy. However, it remains imperative to analyzes the challenges associated with translating DC-based immunotherapies from preclinical models to clinical applications, including scalability, safety concerns, and regulatory issues.

Finally, advanced therapy medicinal products – and most notably cell-based therapies – represent cutting-edge treatments primarily focused on many chronic diseases and significant unmet medical needs. The clinical evidence generated and the quality of these advanced therapies are crucial for their development, approval, and post-marketing stages. In this review, we have made an attempt to outline the current state of clinical development for advanced therapies, highlighting the challenges and discussing potential solutions being considered. The clinical evidence generated and the quality of these advanced therapies are crucial for their development, approval, and post-marketing stages.

Most approved advanced therapy strategies rely on adaptive, small-scale, open-label, uncontrolled, and single-arm pivotal trials. Regulators have shown flexibility in conventional regulatory requirements, particularly for low-prevalence, life-threatening, or severely debilitating conditions. The ongoing advancement of scientific standards aims to ensure consistency in clinical development and reproducibility of knowledge. This progression is essential not only for increasing the evidence base for approval but also for establishing principles that support translational success in this field (Greco et al., 2024).

While there is a growing trend towards adaptive or life-cycle licensing approaches, regulators and global working groups are currently developing new recommendations to foster methodologically robust clinical development. These new guidelines aim to make clinical trials significantly more relevant. The future evolution of clinical development of cell-based therapies remains uncertain, but it is advised that industry stakeholders should understand and apply these recommendations to improve their chances of successful market access (Iglesias-Lopez et al., 2021). The ongoing advancement of scientific standards aims to ensure consistency in clinical development and reproducibility of knowledge. This progression is essential not only for increasing the evidence base for approval but also for establishing principles that support translational success in this field.
